# Life before impact in the Chicxulub area: unique marine ichnological signatures preserved in crater suevite

**DOI:** 10.1038/s41598-022-15566-z

**Published:** 2022-07-05

**Authors:** Francisco J. Rodríguez-Tovar, Pim Kaskes, Jens Ormö, Sean P. S. Gulick, Michael T. Whalen, Heather L. Jones, Christopher M. Lowery, Timothy J. Bralower, Jan Smit, David T. King, Steven Goderis, Philippe Claeys

**Affiliations:** 1 Departamento de Estratigrafía y Paleontología, Universidad de Granada, Granada, Spain; 2 Research Unit: Analytical, Environmental and Geo-Chemistry, Department of Chemistry, Vrije Universiteit Brussel, AMGC-WE-VUB, Pleinlaan 2, 1050 Brussels, Belgium; 3 Laboratoire G-Time, Université Libre de Bruxelles, Av. F.D. Roosevelt 50, 1050 Brussels, Belgium; 4 Centro de Astrobiologia CSIC-INTA, Torrejon de Ardoz, Spain; 5 Institute for Geophysics, Jackson School of Geosciences, University of Texas at Austin, Austin, USA; 6 Department of Geological Sciences, Jackson School of Geosciences, University of Texas at Austin, Austin, USA; 7 Center for Planetary Systems Habitability, University of Texas at Austin, Austin, USA; 8 Department of Geosciences, University of Alaska Fairbanks, Fairbanks, AK USA; 9 Department of Geosciences, The Pennsylvania State University, College town, USA; 10 Faculty of Sciences (FALW), Vrije Universiteit Amsterdam, Amsterdam, The Netherlands; 11 Department of Geosciences, Auburn University, Auburn, AL USA

**Keywords:** Environmental sciences, Planetary science

## Abstract

To fully assess the resilience and recovery of life in response to the Cretaceous–Paleogene (K-Pg) boundary mass extinction ~ 66 million years ago, it is paramount to understand biodiversity prior to the Chicxulub impact event. The peak ring of the Chicxulub impact structure offshore the Yucatán Peninsula (México) was recently drilled and extracted a ~ 100 m thick impact-generated, melt-bearing, polymict breccia (crater suevite), which preserved carbonate clasts with common biogenic structures. We pieced this information to reproduce for the first time the macrobenthic tracemaker community and marine paleoenvironment prior to a large impact event at the crater area by combining paleoichnology with micropaleontology. A variable macrobenthic tracemaker community was present prior to the impact (Cenomanian–Maastrichtian), which included soft bodied organisms such as annelids, crustaceans and bivalves, mainly colonizing softgrounds in marine oxygenated, nutrient rich, conditions. Trace fossil assemblage from these upper Cretaceous core lithologies, with dominant *Planolites* and frequent *Chondrites*, corresponds well with that in the overlying post-impact Paleogene sediments. This reveals that the K-Pg impact event had no significant effects (i.e., extinction) on the composition of the macroinvertebrate tracemaker community in the Chicxulub region.

## Introduction

**Figure 1:**
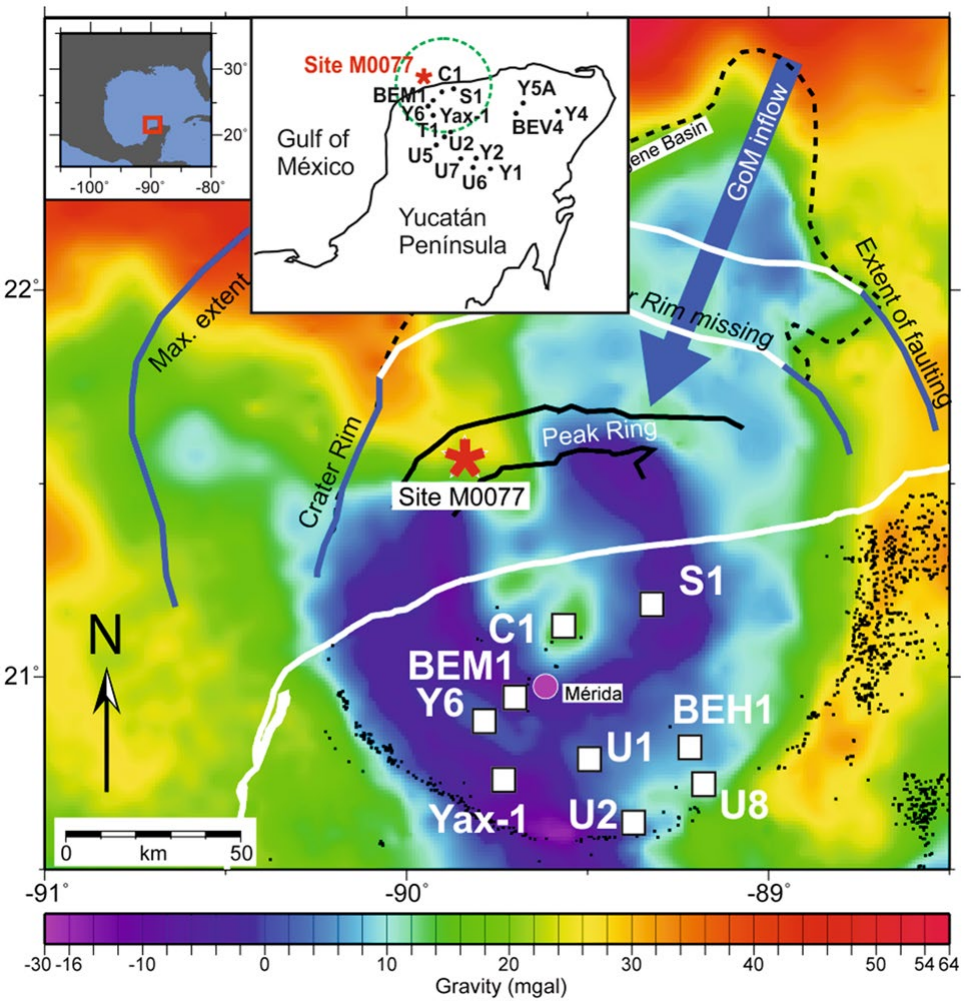
Location of Site M0077 in the Chicxulub Crater on gravity data. Position of selected drilling sites from PEMEX Drilling Program (Y6, C1, S1), UNAM (1 to 8), CPE-UNAM (BEM-1, BEH-1 and BEV-4) and ICDP-UNAM CSDP (Yax-1). The black dots are cenotes and the white line is the coastline. The black dashed line shows the extent of the Cenozoic Chicxulub basin. Modified from Gulick et al.^20^, and Lowery et al.^7^. For detailed location of drilling sites see Gulick et al.^21^.

From April to May 2016, the joint International Ocean Discovery Program (IODP)-International Continental Scientific Drilling Program (ICDP) Expedition 364 at site M0077 recovered an ~ 829 m-long drill core of post-impact sedimentary rocks, impactites, and uplifted basement atop the peak ring of the Chicxulub impact crater, Yucatán Peninsula, México^1,2^ (Fig. 1). The core penetrated Paleogene sedimentary rocks, suevite, melt rock, and granitic basement^1^. The well-preserved, 200-km diameter and ~ 66 Myr old Chicxulub impact crater is one of only three multi-ring impact structures preserved on Earth today. It is also known to be the primary cause to the Cretaceous–Paleogene (K-Pg) mass extinction event that eradicated about 76% of species known from the fossil record, including both terrestrial and marine groups^3,4^. The reconstruction of the recovery of life in a potentially sterilized zone was one of the major research aims of Expedition 364^5–7^. Recent analyses revealed diverse trace fossils observed in the first post-impact deposits, evidence for the rapid recovery of life, and suggesting that the crater became habitable within the first few years after the impact^7–14^. On longer timescales, the timing of the subsequent evolution of the different biota in the crater area is variable during the first stages of the Paleocene, on the order of 10^5^ yr, as revealed by the record of foraminifera, nannoplankton, pollen and spores, and trace fossils^11,14–19^.

Advancing our understanding of the local biological effects of the K-Pg event requires knowledge about what preceded it. Although numerous boreholes were drilled within and around the Chicxulub crater (see Supplementary information), only a few sites retrieved core material that contained the pre-impact sedimentary target rocks, and thus relatively little is known about the biota living at ‘ground zero’ before the impact event. This information mainly refers to microfossils, and comparatively less to macrofossils, including very scarce data on trace fossils^22–27^.

To study the effects of the K-Pg impact event on biota and the regional recovery of life after the impact, information on pre-impact communities is essential. Considering the continuous recovery of post-impact sedimentary rocks from the IODP-ICDP Expedition 364 and data about the life after the impact^7^, a comparison with upper Cretaceous material from the same Site M0077 would be of great benefit to evaluate evolutionary and ecological dynamics of the K-Pg event and its recovery.

The drill site is situated on the Chicxulub peak ring, close to the edge of the excavation cavity, where Cretaceous target rocks were vaporized, melted and extensively redistributed^1^. Therefore, the borehole did not penetrate in situ Cretaceous sediments and thus direct data on pre-impact biota at Site M0077 is lacking. However, clasts of Cretaceous sedimentary rocks within the suevite (see detailed biostratigraphic data from the studied clasts in the Micropaleontology and biostratigraphy section) originate from both excavated material and rip-up of target strata when resurging water passed over the vicinity of the crater and may, thus, be considered to represent the local target rocks^28^. In this way, lithic fragments in the suevite can help reconstruct the missing parts of the target sequence at ‘ground zero’, as well as provide a window into the pre-impact paleoenvironment.

Usually, the fossil record potentially incorporated in suevites is scarce, and often not taken into consideration with respect to other types of information (i.e., geochemical, mineralogical, petrographic, etc.). Data about the pre-impact communities based on impactites mainly refer to microfossils; biostratigraphic characterization and/or interpretations about the microfossil community and pre-impact depositional environments^24,25^, whereas macrofossil remains are comparatively scarce and usually very poorly preserved^24,25,29,30^.

Ichnological information from suevite is nearly absent in the literature. To our knowledge, there are only few reported occurrences of trace fossils in suevite material (e.g., in the Chesapeake Bay impact breccia, USA), and where published these refer to bioturbation in general without any specific ichnotaxa differentiation^31^. However, precise ichnotaxonomical information across the K-Pg boundary is required for a more detailed understanding of the K-Pg mass extinction event and the resilience of macrobenthic trace maker communities.

Ichnological data from the Paleogene sediments from the Expedition 364 M0077A drill core was recently used to characterize bio-events associated with the Chicxulub impact event, and revealed the recovery of life on the seafloor within years of the impact at ground zero^7,11^. Here, we report on trace fossils from carbonate clasts within the suevite from this drill core. This is the first ichnological study conducted on suevite rocks and the main goals are to: (a) reveal the macrobenthic tracemaker community in the Yucatán area before the impact event, (b) assess paleoenvironmental conditions during Late Cretaceous times, and (c) assist in evaluating the effect of the Chicxulub impact event on the local macrobenthic community through comparison of communities occupying ‘ground zero’ prior to (Late Cretaceous) and following (earliest Paleogene) the K-Pg boundary event.

## Geological setting: sedimentology and paleoenvironment

**Figure 2:**
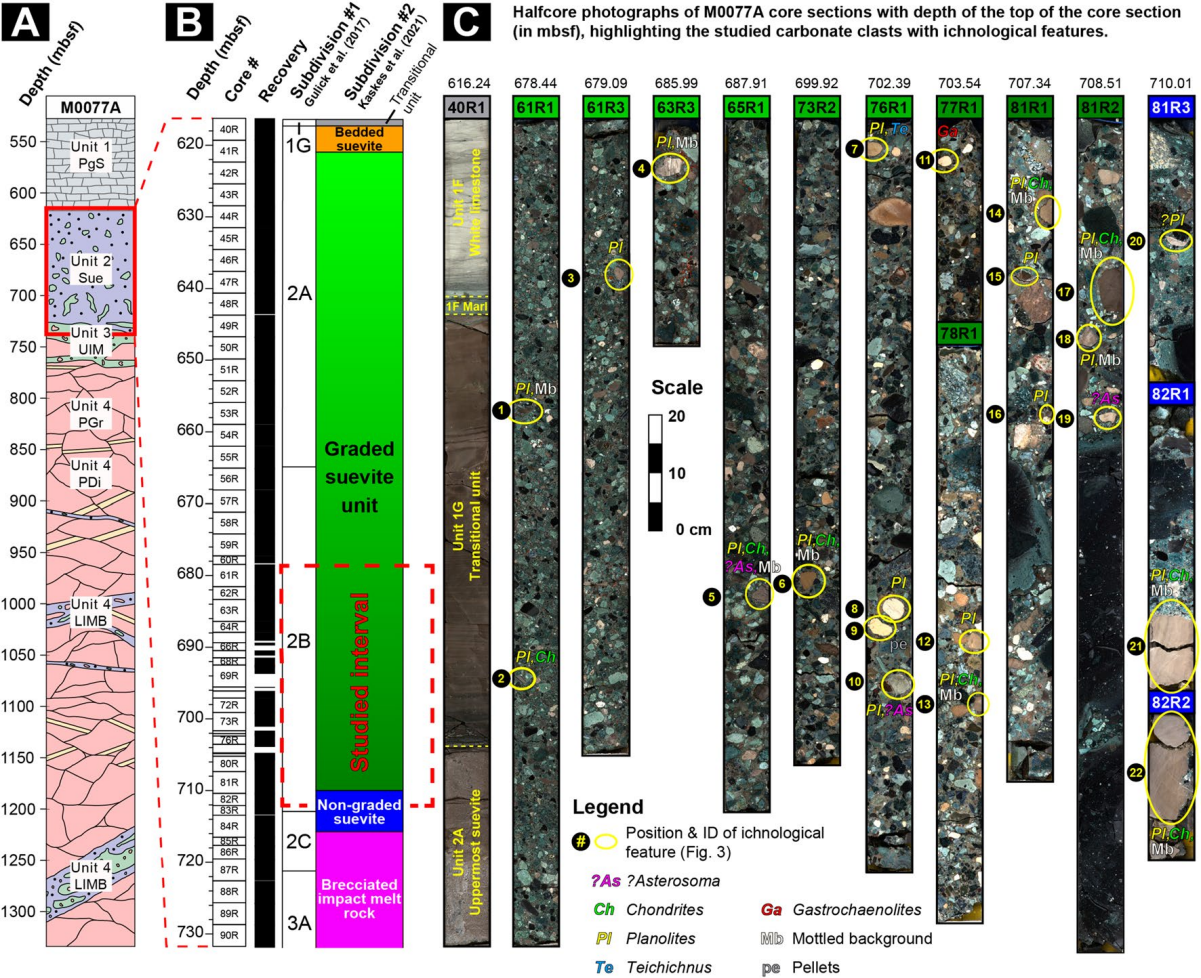
Stratigraphic overview of the IODP-ICDP Expedition 364 M0077A drill core. (**A**) Four main lithological units from Morgan et al.^1,6^. PgS—Paleogene marine sediments; SUE—suevite; UIM—upper impact melt rock unit; LIMB—lower impact melt rock-bearing unit (following de Graaff et al.^33^); GRB—pre-impact granitoid basement; PDI—pre-impact dikes. (**B**) Stratigraphy of the impactite sequence between cores 40 and 90 (∼ 616.5–732 m below sea floor [mbsf]) with the degree of core recovery and the initial subdivision of the sequence by Gulick et al. (Unit 1G; 2A-2C; 3A). Adjacent, an alternative subdivision of this sequence is shown with the three distinct suevite units suggested by Kaskes et al.^12^. The red dashed square indicates the stratigraphic interval in which macroscopic ichnological features are recognized in the suevite clasts. (**C**) Composite halfcore photographs with the core sections of the graded and non-graded suevite unit in which ichnological features are recognized, which are highlighted and labelled (#1–22).

The IODP-ICDP Site M0077 (21.45° N, 89.95° W) is offshore of the Yucatán Peninsula (México), located atop a high-relief portion of the Chicxulub peak ring (Fig. 1)^1,21^. The recovered core was initially subdivided into 4 lithological units^5,6,21^ (Fig. 2A). The upper Unit 1 is a 111.63-m-thick sequence of post-impact hemipelagic and pelagic Paleogene sedimentary rocks, recovered between 505.7 and 617.33 m below sea floor (mbsf). The 75 cm thick lower part (Unit 1G) corresponds to a fine-grained, carbonate-rich “Transitional Unit”. Below follows Unit 2, which is a 104.28-m thick sequence of predominantly melt-bearing, polymict impact breccia (i.e., suevite)^32^ (617.33 mbsf to 721.61 mbsf), of which the greater part was deposited by high-energy oceanic resurge and subsequent oscillations^5,28^. This part of the core is the focus of this study (see "Materials and methods" and Supplementary information). More recently, the suevite sequence was subdivided into three units, distinct in their petrography, sedimentology, and geochemistry^12^: a ∼ 3.5 m thick bedded unit, a ∼ 89 m thick graded unit, and a ∼ 5.6 m thick non-graded unit (Fig. 2B). Below this suevite sequence, a brecciated impact melt rock is encountered with green bands of sparry calcite (schlieren) and rare carbonate clasts, followed by black impact melt rock containing abundant crystalline basement clasts (Unit 3, Gulick et al.^21^) extending to a depth of 747 mbsf. This impact melt and suevite sequence was formed extremely rapidly within the impact basin in < 1 day post-impact^5,12,21,28^. Unit 4 consists of shocked granitoid basement with pre-impact dikes (including dolerites, dacites and felsites), and intercalations of suevite and impact melt rock^5,6,21,33^.

The location of the Chicxulub impact was a carbonate ramp with an average water depth of 600 m^20^, wherein the carbonate ramp deepened from ~ 100 m water depth in the south-southwest to approximately 2 km in the north-northeast^20,34^. The depositional environment during the early Paleocene at Site M0077 corresponded to the upper and/or middle bathyal zone at ~ 600–700 m depth^7^. Sedimentary target clasts from the studied suevite sequence mainly correspond to sediment derived from coastal and shallow-water environments that prevailed throughout much of the paleo-Gulf of Mexico^35^. The sediment clasts of the suevite were initially emplaced by processes initiated by the impact^5,28^, and subsequently deposited in the crater by a powerful ocean resurge^28^, which most probably entered the Chicxulub crater through a N-NE gap in the outer rim^5,12,20,21,28^ (Fig. 1).

Previous work on the Cretaceous part of the Yucatán’s target stratigraphy, based on outcrop studies in both México^24^ and in adjacent northern Belize^36,37^, indicated that shallow-water carbonate facies of the target Yucatán Group show similarities with the limestone clasts in the suevite (see Ormö et al.^28^, for a recent analysis). The sedimentary, (upper) target carbonates could be further subdivided into two main groups for more information of their provenance, the ‘upper target I’ and the ‘upper target II’ carbonates^28^. The ‘upper target I’ carbonates likely derive from the upper part of the Yucatán Group, which is equivalent to the informal Barton Creek Formation in Belize and the Campur Formation of Guatemala, with a Campanian–Maastrichtian age^36^, and ‘upper target II’ likely come from the lower Yucatan Group, which is equivalent to the informal Yalbac formation of Belize and of the Coban Formation of Guatemala^36^. Recent analysis of lithological features and carbonate rock textures of the Barton and Yalbac formation supported that interpretation that target carbonates derived from these formations^95^. Therefore, the trace-fossil bearing carbonate clasts from Hole M0077A likely originated from the upper part of the Yucatán Group target. In the Tabasco-Chiapas-Campeche region, the impact-generated limestone megabreccia of the Guayal and Bochil K-Pg sections also contains foraminifera suggesting a Maastrichtian age^39^.

In the recovered Yax-1 core from the annular trough of the Chicxulub impact structure (Fig. 1), six suevitic units were differentiated in a ∼ 100 m thick impactite sequence, revealing a mixture of late Campanian to early Maastrichtian nannofossil assemblage in the uppermost three suevite units^40,41^. The mixed nature of reworked Campanian to Maastrichtian microfossils, together with lithic fragments and impact derived materials in the suevite of units 2 and 1 in Yax-1 core is similar to the K-Pg boundary “cocktail” deposits in the Gulf of Mexico^42^. The Late Cretaceous biostratigraphic age range found for the studied carbonate clasts within the IODP-ICDP Exp. 364 M0077A core (see detailed biostratigraphic data from the studied clasts in the Micropaleontology and biostratigraphy section) agrees, in part, with observations of planktic foraminifera within the matrix throughout the entire suevite sequence from the same drill core^12^ and from carbonate clasts within Unit 2A^21^. In addition, rare and moderately to poorly preserved nannofossils were reported in the upper part of the M0077A graded suevite between 619.27 and 677.22 mbsf that indicate a Late Cretaceous stratigraphic range^21^.

## Results

### Biogenic sedimentary and bioerosion structures in the suevite.

Suevite from Hole M0077A, referred to as Unit 2^21^, is characterized by a clastic matrix and is dominated by vitric and microcrystalline impact melt rock clasts and fragments from the sedimentary cover and the crystalline basement, the latter displaying varying degrees of shock metamorphism^12,21^. Clasts from target sedimentary lithologies in the suevite mainly include carbonates, both as primary, fossil-bearing clasts, and as altered carbonate clasts, and rare siltstone and chert clasts^12,28^. Isolated Cretaceous planktic foraminifera are present throughout the entire 100 m thick suevite sequence, although the non-graded suevite unit and the bedded suevite unit show more abundant planktic foraminifera in the matrix compared to the graded suevite unit^12^.

Biogenic sedimentary structures are quite common and relatively diverse within the carbonate clasts of the suevite in the M0077A drill core. The ichnological analysis of the entire suevite at Site M0077 reveals biogenic sedimentary structures in core segments from Unit 2B (55R_003-11 cm to 83R_001-75 cm), in particular from core segments between 61_R_002 to 82_R_002 (see Supplementary information and Fig. 2B,C), between core depths ∼679—711 mbsf. Core segments belonging to Unit 2A (40R_1-109.4 cm to 55R_3-11 cm) contain small clasts (in general < 1 cm), and those from Unit 2C (83R_1-75 cm to 87R_2-90 cm), show mainly recrystallized carbonate clasts, wherein biogenic structures can rarely be recognized.

**Figure 3:**
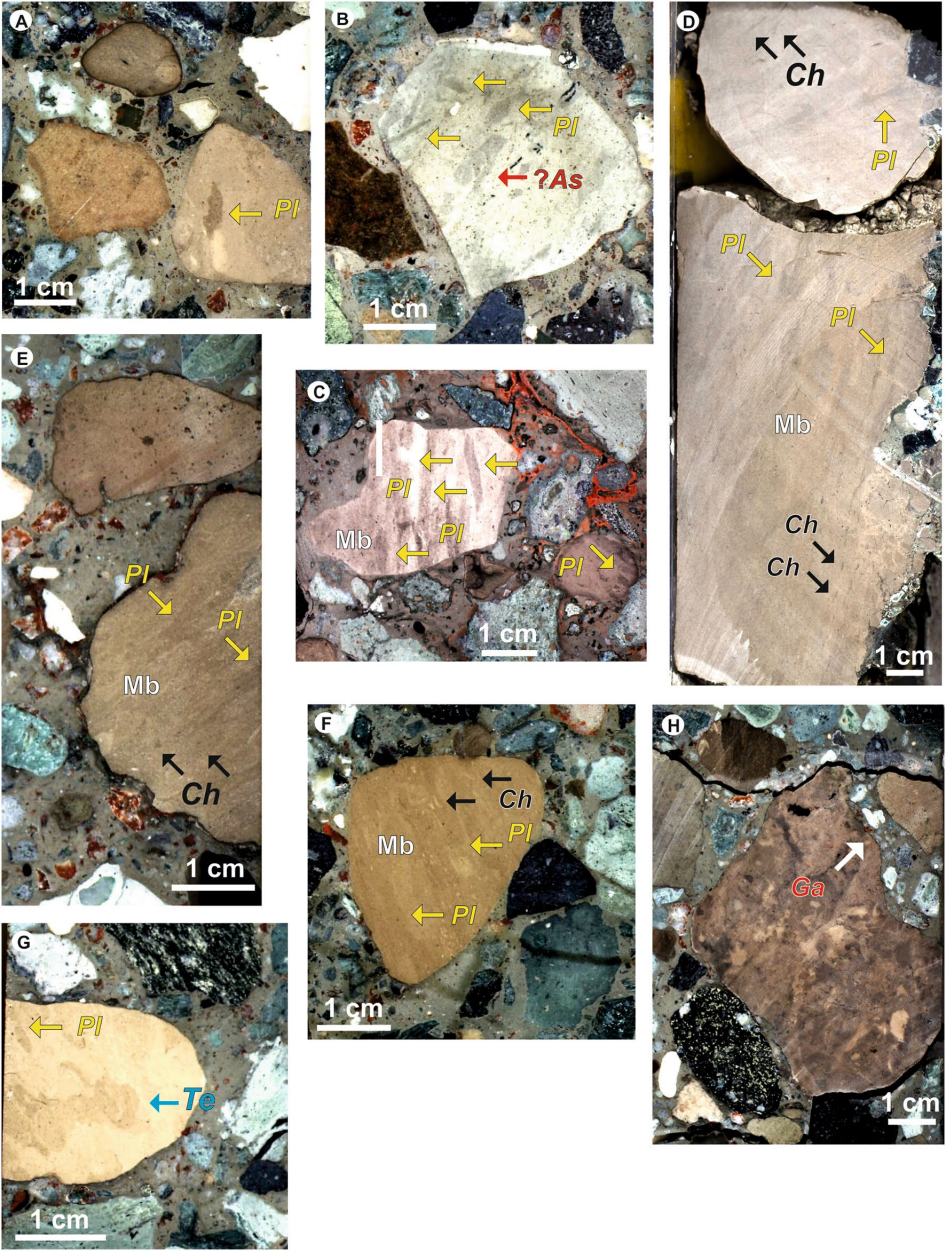
Close-up views of selected trace fossils from Hole M0077A at IODP-ICDP Expedition 364. (**A**) Probable deformed *Planolites* (*Pl*); label 12. (**B**) *Planolites* (*Pl*) and ?*Asterosoma* (?*As*); label 10. (**C**) *Planolites* (*Pl*) and Mottled background (Mb); label 4. (**D**, **E**, **F**) *Planolites* (*Pl*), *Chondrites* (*Ch*) and Mottled background (Mb); labels 22, 14, and 6, respectively. (**G**) *Planolites* (*Pl*) and *Teichichnus zigzag* (*Te*); label 7. Labels refer to Fig. 2.

The biogenic sedimentary structures are mainly preserved in light brown carbonate clasts, usually between 1 and 5 cm in size and locally up to 20 cm, observed in several core segments of the lower part of the graded suevite unit and the upper part of the non-graded suevite unit (Figs. 2, 3; Kaskes et al.^12^). The most abundant trace fossil is *Planolites*, registered in the 86% of the studied clasts, which appears in almost all the studied core segments as circular to subcircular, cylindrical, tubular forms of a variable size (diameter 2–4 mm, length 5–20 mm; Figs. 2C, 3A–G, S1). The record of other ichnotaxa is frequent as in the case of *Chondrites* (36% of the clasts), or sporadic for ?*Asterosoma* (14% of the clasts) and *Teichichnus* (*T. zigzag*) (5% of the clasts) (Fig. S1). ?*Asterosoma* are observed as bulbous forms, in light-gray and brown carbonate clasts usually together with *Planolites* (Figs. 2C, 3B). *Chondrites* are mainly observed in brown carbonate clasts (Figs. 2C, 3D–E,F), usually on a mottled background, as short tubes or circular to elliptical spots, 1–2 mm in diameter. One specimen of *Teichichnus zigzag* is observed in a light brown carbonate clast (Figs. 2C, 3G), appearing as a wall-like spreite (e.g., laminated biogenic structure) structure with a zig-zag vertical section, 2–3 cm in length and 0.5 to 1 cm in diameter. Biodeformational structures as those showing undifferentiated outlines and the absence of a defined geometry producing a mottled fabric are observed in several clasts (45%). In some carbonate clasts (Figs. 2C, 3C–F), the mottled background is crosscut by discrete *Chondrites* and *Planolites*. A bioerosion structure appearing as a single cylindrical form, less than 5 mm long, observed in a light brown clast (label 11 in Fig. 2C), is designated as probable *Gastrochaenolites*. Ichnodiversity is variable between the studied clasts, in most of cases showing one (52%), or two (43%) different ichnotaxa. The most frequent assemblage (29% of the clasts) consists of *Planolites* and *Chondrites* overprinting a mottled background. Amount of bioturbation (as Bioturbation Index) into the studied clasts is variable from 1 to 6, considering the mottled background, or from 1 to 4 considering only trace fossils. According to the above, trace fossil assemblage is dominated by trace fossils belonging to shallow and middle tiers, reflecting the activity of tracemakers just below the seafloor, and a few centimeters deep within the substrate.

### Micropaleontology and biostratigraphy

**Figure 4:**
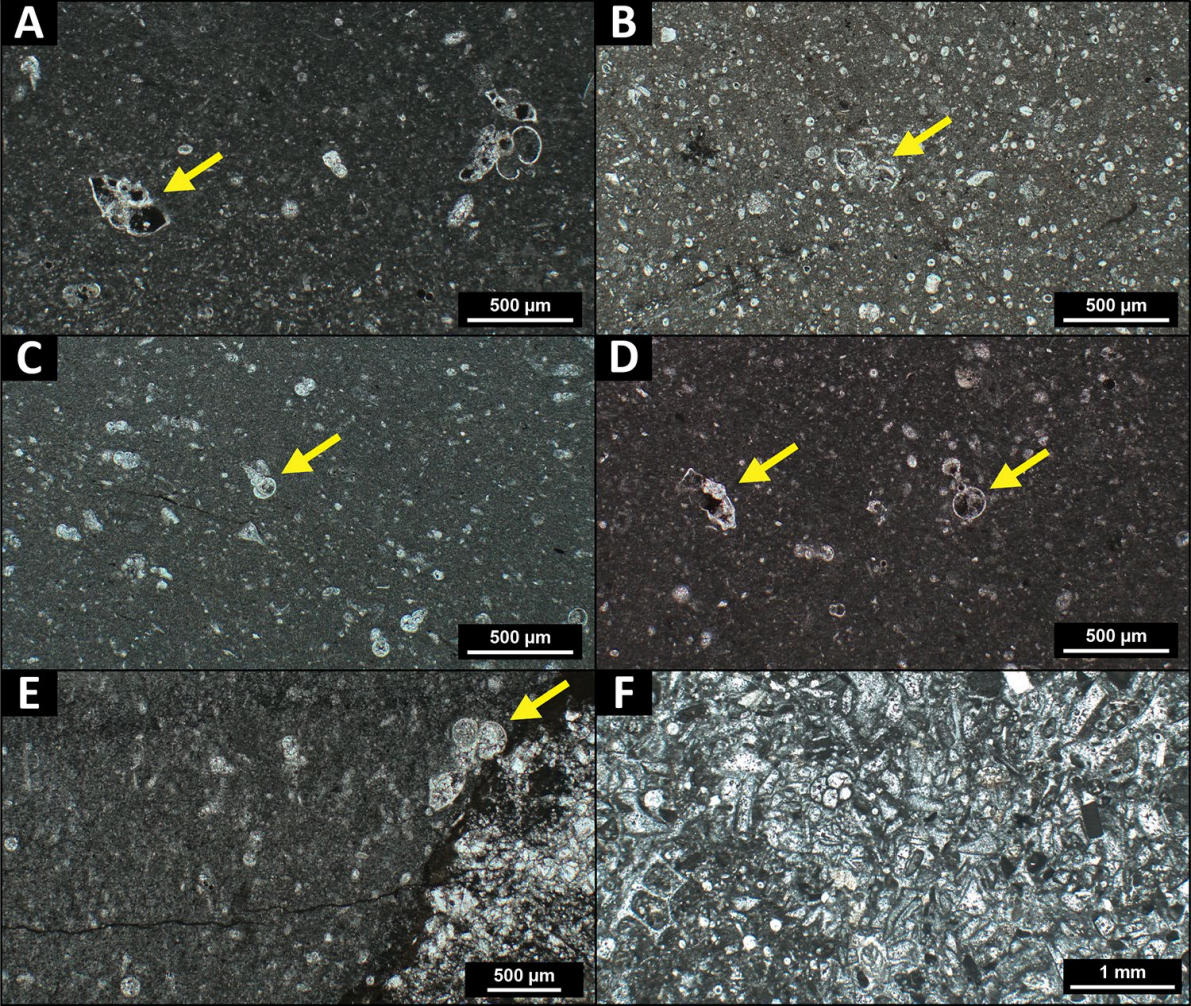
Representative PPL microphotographs of M0077A suevite carbonate clasts that yield ichnological features. Age-determinant planktic foraminifera are indicated with yellow arrows. (**A**) Pelagic wackestone with *Globotruncanita stuarti* (core 77_1_6.5; 703.61 mbsf). (**B**) Pelagic packstone with abundant calcispheres and *Muricohedbergella* sp. (core 81_2_27; 708.78 mbsf). (**C**) Wackestone with *Planoheterohelix* sp. (core 65_1_79; 688.70 mbsf). (**D**) Pelagic wackestone with* Globotruncana* sp. and *Globigerinelloides* sp. (core 77_1_6.5; 703.61 mbsf). (**E**) Wackestone with *Rugoglobigerina* sp. (core 61_3_25; 680.75 mbsf). (**F**) Coarse packstone with shallow marine carbonate debris rich in larger benthic foraminifera (core 76_1_87; 703.26 mbsf).

A selection of the suevite carbonate clasts containing ichnological features was investigated for micropaleontological content to assess their depositional environment and geological age (Supplementary Table 1). The majority of the studied clasts are wackestones and packstones with a micritic matrix and a variable fossil content with grains < 2 mm, including shell fragments, calcispheres, calcareous nannoplankton and planktic and benthic foraminifera of variable preservation (Fig. 4). The planktic foraminifera (in general 100–500 µm in size) include taxa such as *Globigerinelloides* sp. (Valanginian to Maastrichtian in age), *Globotruncanita stuarti* (Campanian–Maastrichtian), *Globotruncana* sp. (Coniacian-Maastrichtian), *Muricohedbergella* sp. (Albian-Maastrichtian), and *Planoheterohelix* sp. (Cenomanian–Maastrichtian) (Smit^43^, and references therein). Larger benthic foraminifera (300 µm up to several millimeters in size) range from miliolid foraminifera such as *Quinqueloculina* sp. to orbitoidal foraminifera such as *Orbitoides* sp. (Santonian-Maastrichtian) (Alegret and Thomas^44^ and references therein). These foraminiferal assemblages in the studied carbonate clasts yield a Late Cretaceous age (∼ 100.5–66 Ma), ranging from the Cenomanian until the end of the Maastrichtian (Supplementary Table 1). Nannofossil analysis of selected target clasts reveals abundant *Braarudosphaera* spp., together with *Watznaueria barnesiae*, *Cyclagelosphaera reinhardtii*, and *Cribrosphaerella* spp. Age diagnostic species include *Eiffellithus eximius* (Turonian-Campanian), *Aspidolithus parcus* (Campanian), *Lithraphidites quadratus* (Maastrichtian), and *Micula murus* (upper Maastrichtian) (Burnett^45^ and references therein), also indicating a generic Late Cretaceous age.

## Discussion

### The pre-impact macrobenthic tracemaker community in the Chicxulub area

The ichnological record from the Chicxulub peak-ring suevite provides unique insights on the macrobenthic tracemaker community living at ‘ground zero’ during the Late Cretaceous, especially given the scarcity of other ichnological information from the Yucatán area (see Supplementary information). Scarce ichnological data was documented far (more than 1.000 km) from the Chicxulub impact crater area, as from the Navarro-Taylor Sequence, the last unit deposited in the Gulf of Mexico prior to the Chicxulub impact event^46^. Campanian Taylor sandstones from the Serbin field (southern Texas) commonly contain *Ophiomorpha* and irregular, burrowed tops; *Planolites* typically overlie the tops of these sandstones. In addition, *Cruziana* ichnofacies was reported suggesting that the depositional environment was shallow marine and close to the shoreline^26^. In northern Louisiana, Shellhouse^27^ presented a description of a cored succession including contacts between the K-Pg boundary unit and the underlying chalk of the Navarro Group NT supersequence^47^. The cored succession can be subdivided into three main units, indicating the presence of abundant *Thalassinoides* and *Chondrites* in the lower unit, *Thalassinoides* and *Helminthopsis* in the middle unit, and rare *Helminthopsis* in the upper unit. In Northeastern Mexico, an abundant and diverse Late Cretaceous ichnofauna, with *Chondrites*, *Ophiomorpha*, *Planolites* and *Zoophycos* was documented^48^.

The ichnological data can help to assess paleoenvironmental, paleoecological and depositional conditions in the Yucatán area. Moreover, such data also serve as a reference in comparisons with the life forms inhabiting the crater after the drastic paleoenvironmental changes caused by the Chicxulub impact. The presence of a mottled background in several clasts (Fig. 3C–F) reveals the activity of tracemakers at the sediment water interface or just below the sea floor, in softground, causing the complete destruction of primary structures by the shallowest burrowing organisms^49^. This observation points to general paleoenvironmental conditions on the sea bed favorable for diverse life forms. Thus, oxic bottom and pore waters in the upper part of the bioturbated zone, and available organic matter, allowed for the development of a shallow tier macrobenthic community. *Planolites* (Fig. 3A–G) is a facies-crossing form, featuring actively filled burrows, interpreted as a grazing trace reflecting a combination of locomotion and feeding (pascichnion) most likely produced by soft bodied invertebrates (i.e., wormlike animals) in diverse environments^50–52^. *Planolites* is usually interpreted as a shallow tier trace fossil, and its dominance or exclusiveness can be related to environmental parameters favorable for the tracemakers (i.e., oxic and nutrient rich conditions), supporting the interpretation of the origin of the mottled background. *Asterosoma* (Fig. 3B) is commonly considered as a feeding trace (fodinichnion) produced by polychaete and other worms or suspension-feeding animals, such as certain crustaceans^53^. This trace is frequently registered in a wide range of marine environments, from paralic to deep-marine settings, commonly associated with well-oxygenated conditions^53^. *Chondrites* (Fig. 3D–F) shows a wide range of morphologies, and many organisms are proposed producers, such as annelids (e.g., polychaetes), siphunculans, or bivalves^53^. *Chondrites* may be considered a facies-crossing form, registered in a variety of facies and environments. However, it is usually interpreted as a good indicator of dysoxic settings, where the dissolved oxygen in bottom and pore waters is between 0.2 and 1 ml/l^53^, frequently observed in deep-marine environments. The behavior of the tracemaker is variable, possibly resulting from subsurface deposit-feeding behavior, suspension-feeding, detritus-feeding on the sea floor, or it could even be interpreted as a chemosymbiotic organism; the trace is produced preferentially in fine-grained softgrounds and locally in relatively cohesive substrates^53^. *Teichichnus zigzag* (Fig. 3G) is associated with the activity—mainly suspension and deposit feeding— of worm-like animals (e.g., annelids), but also arthropods (e.g., crustaceans) and bivalves, preferentially in silty and muddy sand softgrounds^53^. *Teichichnus zigzag* is usually observed in siliciclastic, marginal-marine to shallow-marine settings. *Teichichnus zigzag* can be interpreted as an equilibrium trace due to changes in sedimentation and erosion as occur in the upper shoreface, tidal flat, delta plain, etc.^53^. The presence of a bioerosion structure, probable *Gastrochaenolites* (Fig. 3H), is related to consolidated or cemented sedimentary materials in proximal areas. These settings such as firmgrounds or hardgrounds, are colonized by boring bivalves^54,55^.

### The late cretaceous Yucatán sea

Ichnological data reveal the presence of a diverse macrobenthic tracemaker community in the Yucatán area during the Cenomanian to the Maastrichtian, including soft bodied organisms such as annelids and shelly animals including crustaceans and bivalves. These organisms mainly colonized softgrounds at the sediment water interface or just below the sea floor, as a feeding activity. Bioerosion structures in firmground-hardgrounds were likely formed by bivalves. This diverse tracemaker community points towards a variety of habitats and paleoenvironmental conditions ranging from coastal to shelfal and more pelagic environments. In this context, distance to shore, suggested to be 800 km away^5^ based on a paleogeographic reconstruction of shorelines^56,57^, and bathymetry are important parameters to be considered. Most of the observed biogenic structures, including the probable *Gastrochaenolites*, were formed in a wide range of paleoenvironments, but mainly in shelfal settings. However, *Chondrites* is usually registered in deeper environments than the rest of the recognized ichnotaxa, which supports variability in the paleowater depth related to the initial formation of the sedimentary clasts before they brecciated, transported and became part of the suevite sequence. Thus, deep-water carbonate facies were probably incorporated into the M0077A suevite sequence, as evidenced with large quantities of planktic foraminifera and nannofossils in the lower part of the suevite.

### The Chicxulub impact event and its effect on macrobenthic biota

The registered softground trace fossil assemblage consisting of dominant *Planolites*, frequent *Chondrites*, and very rare ?*Asterosoma* and *Teichichnus* (*T. zigzag*), is quite similar (mainly consisting of *Planolites* and *Chondrites* and rare *Palaeophycus*) to that observed in the first phase of diversification (diversification I)^14^ after the initial recovery that occurred within years to decades after the impact event, as recorded in the Transitional Interval in the M0077A core (Fig. 2)^5,7,11,13^. Particularly, the most frequent assemblage (29% of the clasts) consisting of *Planolites* and *Chondrites* overprinting a mottled background registered in the Upper Cretaceous clasts is similar to the assemblage observed at the early Paleocene sediments^14^ (Fig. S1). Moreover, ichnotaxa from the Upper Cretaceous clasts show similar dimensions than those from the early Paleocene. However, abundance of traces (as BI) is higher at the Upper Cretaceous clasts^14^ (Fig.S1). Both trace fossils assemblages, from the Upper Cretaceous clasts and from the early Paleocene sediments, are more diverse, abundant and consisting of larger traces, that the trace fossil assemblage corresponding to the initial recovery just after the K-Pg event^14^ (Fig. S1). In this initial recovery phase, trace fossil assemblage is low abundant (BI ~ 1), and mainly consists of smaller *Planolites* (< 4 mm size), and very scarce *Chondrites* (< 1 mm size)^14^ (Fig. S1).

Even though the ichnological information in this study is fragmentary as it is derived from target clasts, some interpretations with respect to the ecological consequences of the impact event on the macrobenthic tracemaker community can be made. The similarity in trace fossil assemblage and in size of traces in Upper Cretaceous and early Paleocene sediments in the crater area agrees with the absence of significant effects (i.e., extinction) of the Chicxulub impact event on the global marine macrobenthic tracemaker community, and the rapid recovery of the macrobenthic tracemaker community, as previously observed in distal K-Pg boundary sections such as in Spain and Denmark^4,49,50,52,58–62^. Pre-impact macrobenthic tracemaker community during Upper Cretaceous, dominated by the shallow and middle tiers, is similar, but more abundant, that the post-impact community registered during the early Paleocene. In between, the initial recovery phase occurred within several years after the K-Pg boundary impact, is characterized by a less diverse and scarcer trace fossil assemblage^14^. This comparatively minor disruption and the rapid return to the pre-extinction macrobenthic tracemaker community of the impact site^6^ could be related to the existence of less affected habitats outside the crater and then migration of these biota into the crater when favourable conditions were re-established. This finding implies rapid recolonization of the impact site^7,11^ by the macrobenthic tracemaker community. Trace fossils of this community do not appear at the base of the Transitional Unit but rather within the upper ~ 30 cm of it, which likely represents a few years after impact^5,9,13^. This suggestion is in line with other data which suggest that the crater seafloor may not have yet been habitable for a brief period after the impact^7^.

## Materials and methods

The entire 98.3 m thick suevite unit (617.33 mbsf, 40R-1-109.4 cm to 715.6 mbsf, 84R-3-78 cm) in core M0077A was preliminary studied. This unit was subdivided into Units 2A, 2B and 2C^21^ based on sedimentary features and matrix composition. Then, this interval was divided into a bedded suevite, graded suevite and non-graded suevite unit that are a distinct in petrography, geochemistry, and sedimentology^12^. Unit 2B, from 664.52 mbsf (55R-3-11 cm) to 712.84 mbsf (83R-1-75 cm) contains abundant clasts in which biogenic structures and/or fossils were observed due to a generally larger clast size than in the Unit 2A above (Kaskes et al.)^12^; this was the selected interval studied in detail. The suevite at Site M0077 was studied for ichnological features through a detailed visual examination of high-resolution digital images of the 83 mm wide archive half cores, including Core overview, Line Scan, and X-ray computed tomography (CT, CTA, and CTD) images, using a digital image methodology^63–65^ (see Supplementary information). This processing enhanced the structures of interest in the images allowing better recognition of variable types of biogenic structures, as biogenic sedimentary structures and bioerosion structures, and the ichnotaxonomical classification of discrete trace fossils^66–68^ (see Supplementary information). In addition, biostratigraphic analyses were performed on ten polished 30 µm thin sections of the investigated carbonate clasts by means of a micropaleontological assessment focusing on the taxonomy of planktic and benthic foraminifera, and nannofossil analysis.

## Supplementary Information

Supplementary Information 1.

Supplementary Information 2.

Supplementary Information 3.

## Data Availability

All data generated or analysed during this study are included in this published article [and its supplementary information files]. The datasets generated and/or analysed during the current study are not publicly available due to size restrictions, but are available from the corresponding author on reasonable request.
