# Supplementary material for: Life before impact in the Chicxulub area: unique marine ichnological signatures preserved in crater suevite
Source: Sci Rep. 2022 Jul 5;12:11376. doi: 10.1038/s41598-022-15566-z (PMC9256630; doi:10.1038/s41598-022-15566-z)
Supplement: Supplementary file 1 — Supplementary Information 1. [file 41598_2022_15566_MOESM1_ESM.docx]

Supplementary Information

**Life before impact in the Chicxulub area: Unique marine ichnological signatures preserved in crater suevite**

Francisco J. Rodríguez-Tovar^1*^, Pim Kaskes^2,3^, Jens Ormö^4^, Sean P.S. Gulick^5,6,7,^, Michael T. Whalen^8^, Heather L. Jones^9^, Christopher M. Lowery^5^, Timothy J. Bralower^9^, Jan Smit^10^, David T. King Jr.^11^, Steven Goderis^2^, Philippe Claeys^2^

^1^Departamento de Estratigrafía y Paleontología, Universidad de Granada, Spain

^2^Research Unit: Analytical, Environmental & Geo-Chemistry, Department of Chemistry, Vrije Universiteit Brussel, AMGC-WE-VUB, Pleinlaan 2, 1050 Brussels, Belgium

^3^Laboratoire G-Time, Université Libre de Bruxelles, Av. F.D. Roosevelt 50, 1050 Brussels, Belgium

^4^Centro de Astrobiologia CSIC-INTA, Torrejon de Ardoz, Spain

^5^Institute for Geophysics, Jackson School of Geosciences, University of Texas at Austin, USA

^6^Department of Geological Sciences, Jackson School of Geosciences, University of Texas at Austin, USA

^7^Center for Planetary Systems Habitability, University of Texas at Austin, USA

^8^Department of Geosciences, University of Alaska Fairbanks, Fairbanks, AK, USA

^9^Department of Geosciences, The Pennsylvania State University, USA

^10^Faculty of Sciences (FALW), Vrije Universiteit Amsterdam, Netherlands

^11^Department of Geosciences, Auburn University, Auburn, AL, USA

* Correspondence to: fjrtovar@ugr.es

**Supplementary Information**

Supplementary Note

Supplementary Table 1

**Supplementary Note**

*Previous Yucatán drill core data*

Several drillings have been conducted on the Yucatán Peninsula in Mexico prior to IODP-ICDP Expedition 364, corresponding to the PEMEX, UNAM, CSDP-ICDP and CPE-UNAM Drilling Programs (Fig. 1). During the 1950s and 1960s, the PEMEX oil exploration company drilled a sequence of Mesozoic and Cenozoic carbonate platform sedimentary rocks, as well as impact melt rock and impact breccias^7,8,10,15^. Mesozoic (pre-impact) sedimentary rocks and impact breccias were drilled in boreholes Ticul-1 (T1), Yucatán-2 (Y2), Yucatán-5A (Y5A), Yucatán-1 (Y1) and Yucatán-4 (Y4), and impact breccias without intact Mesozoic stratigraphy were drilled in boreholes Chicxulub-1 (C1), Sacapuc-1 (S1) and Yucatán-6 (Y6)^21,22,30,31^. In 1994 and 1995, the UNAM program drilled eight shallow boreholes (UNAM-1 to UNAM-8^69,70^, all of them coring the basal Paleogene carbonate sequence^71-75^, and three of them —Santa Helena UNAM-5, Peto UNAM-6 and Tekax UNAM-7— recovering impact breccias with carbonate and anhydrite clasts^74^. As part of the joint Chicxulub Scientific Drilling Project and International Continental Drilling Project (CSDP-ICDP), the Yaxcopoil-1 (Yax-1) borehole was drilled in 2001/2002. This borehole has extensively been studied, showing a thick succession of Paleogene sedimentary rocks, impactites (including suevites), and a megablock of Cretaceous carbonates and evaporites^76-79^. Finally, the CPE-UNAM program drilled three boreholes, BEM-1, BEH-1 and BEV-4, recovering impact breccia and a carbonate sequence (mega-breccia unit or pre-impact Cretaceous target sequence) in the eastern sector of the Chicxulub crater^75^.

*Previous paleontological data in the suevite rocks at the Chicxulub holes*

In view of the various core holes drilled in the Chicxulub crater, scarce paleontological information has been derived from the suevite rocks, restricted only to the PEMEX (i.e., Y4, Y6) and CSDP-ICDP Yax-1 cores. Paleontological data mainly refer to microfossils (i.e., foraminifera), rarely to macrofossil, and are absent for ichnofossils.

In the PEMEX cores, benthic and planktic foraminifera were found in Mesozoic target materials, allowing characterization of middle and upper Cretaceous (Albian to Maastrichtian) sediments mainly deposited in a shallow-water carbonate platform^21,22,30,31^. The impact breccia in the Yucatán 6 (Y6) is variable and complex in terms of composition and stratigraphy, containing several types of carbonate fragments (mainly within the upper, carbonate-rich suevite)^31^ from the uppermost part of the target sequence, with abundant planktic and benthic Maastrichtian foraminifers^22^, but also mollusk, algal and sponge fragments^30,31^. This assemblage indicates a shallow water carbonate platform^31^, and a water depth prior to the impact event of a few tens of meters at most^23^. After the impact, abundant occurrences of Danian planktic foraminifera were interpreted to reveal a deeper depositional setting^23^.

In the Yax-1 core, fossils have been identified in the Cretaceous megablock and Paleogene sediments, including observations of bioturbation^23,76,77,79-81^. The impactite sequence in Yax-1 also revealed the presence of fossil remains^23,82^, as abundant microscopic calcareous shells and foraminifera within carbonate clasts or as isolated components suspended in the matrix of impact-related breccias^82^. Based on the presence of carbonate clasts and foraminifera, it was proposed that the uppermost suevite interval (unit 1)^82^ was reworked by high-energy sedimentary processes in the form of a turbulent resurge of seawater into the impact basin^39^, mixing reworked unshocked debris with shocked material. A detailed paleontological analysis is presented in Stinnesbeck et al.^23^, indicating the presence of micro- and macroinvertebrate fossils in Cretaceous and Paleogene sediments, including planktic and benthic foraminifera, calcispheres, bivalves, and even trace fossils such as *Chondrites* and *Thalassinoides* were found in the Cretaceous megablock and in the earliest Danian sediments. Unit H^23^, corresponding to the suevite breccia, contains clasts that include isolated fossils as benthic foraminifers and bivalves, but no ichnological features were identified.

Recently, in the basal graded suevite unit of the core M0077A, abundant large limestone clasts containing a variety of upper Cretaceous large benthic foraminifers, miliolids, and rudist fragments have been found^12,26^ (Fig. 2). The impact on the Yucatán Peninsula took place in a marine setting with a variable water depth, from the estimated 600 m deep at the impact site to ~2 km deep toward the north and shallower settings toward the south^52^ (see discussion in Kaskes et al.^12^). In addition, an increase in the amount of (upper) Maastrichtian foraminifers in the bedded suevite unit was noted^73^, indicative of reworking due to in-crater seiche activity after the main ocean resurge^12^.

**Methods**

Ichnological analysis in cores is challenging owing to the properties of the cores, such as the limited size and restricted exposed surface, the almost exclusive availability of two-dimensional core slab images, and the usual absence of complete (3D) structures, making the characterization of trace fossils much more difficult^49,63,84,85,91^. In this study, high-resolution, two-dimensional, digital line-scan images of the archive half from the M0077A drill core were used^26^, these color photographs are available at: <http://publications.iodp.org/proceedings/364/EXP_REPT/CORES/IMAGES/>. To improve visibility, the images were processed (when necessary) using a digital image methodology by adjusting such parameters as image levels, brightness and vibrance^86,87^, previously successfully applied in marine cores^64,65,84-90^. The proposed technique is applied upon the digital images using the software Adobe Photoshop CS6 (Adobe Systems, San Jose, CA, USA). The method is based on the modifications in several image features as levels, brightness and vibrance: a) levels were applied to reduce the histogram and improve the contrast, b) brightness adjustment was applied to increase the contrast and control the brightness, and c) vibrance was used to make slight modifications. The application allows enhancing visibility of weakly observed trace fossils in the high-resolution digital images, including particular ichnological features as internal structures.

Ichnological analysis has included detailed information on major ichnological attributes, such as ichnodiversity, relative abundance of ichnotaxa, ichnological features (i.e., dimensions), cross-cutting relationships and tiering structure^89^. Biodeformational structures were characterized by undifferentiated outlines and the absence of a defined geometry, producing a mottled fabric, while trace fossils show differentiated outlines and characteristic shapes^66,67^. Within the trace fossils, a distinction is made between bioturbation structures as those produced by the activity of an organism upon, or within, an unconsolidated substrate, and bioerosion structures comprising those structures produced mechanically or biochemically by an organism in rigid substrates. The conducted differentiation between bioturbation structures and bioerosion structures was based on the general morphology of the structure in the lithic clasts in the suevite sequence. Ichnotaxonomical classification of trace fossils was largely based on the overall shape and the presence of certain diagnostic criteria or ichnotaxobases, including size, branching, or internal structure^49,63,64^. Quantification of ichnodiversity and abundance of differentiated ichnotaxa have been calculated in any of the studied clasts. Amount of bioturbation has been characterized and referred to as Bioturbation Index^92,93^ considering discrete trace fossils and the mottled background or overlooking the mottled background^94^.

**Results**

The biogenic structures are mainly preserved in light brown carbonate clasts observed in several core segments of the graded and non-graded suevite units^12^. A total of 22 clasts with trace fossils have been recognized (Fig. 2C), which are listed below. An ichnologic, petrographic, micropaleontological and biostratigraphic description of a selection of these clasts is highlighted in Table S1.

364_77_A_061_R_002: Clast 1 (49-51 cm from top; 679.58 mbsf): Mottled background, *Planolites*, Clast 2 (95-96 cm from top; 680.04 mbsf): *Chondrites*, *Planolites*.

364_77_A_061_R_003: Clast 3 (26-28 cm from top; 680.76 mbsf): *Planolites*.

364_77_A_063_R_003: Clast 4 (7-10 cm from top; 686.06 mbsf): Mottled background, *Planolites*.

364_77_A_065_R_001: Clast 5 (79-83 cm from top; 688.70 mbsf): Mottled background, ?*Asterosoma*, *Chondrites*, *Planolites*.

364_77_A_073_R_002: Clast 6 (77-80 cm from top; 700.69 mbsf): Mottled background, *Chondrites*, *Planolites*.

364_77_A_076_R_001: Clast 7 (4-6 cm from top; 702.43 mbbsf): *Planolites*, *Teichichnus*, Clast 8 (84-87 cm from top; 703.23 mbsf): *Planolites*, Clast 9 (87-90 cm from top; 703.26 mbsf): pelloids, Clast 10 (97-100 cm from top; 703.36 mbsf): ?*Asterosoma*, *Planolites*.

364_77_A_077_R_001: Clast 11 (7-9 cm from top; 703.61 mbsf): *Gastrochaenolites*-like.

364_77_A_078_R_001: Clast 12 (50-52 cm from top; 704.24 mbsf): *Planolites*, Clast 13 (60-63 cm from top; 704.34 mbsf): Mottled background, *Chondrites*, *Planolites*.

364_77_A_081_R_001: Clast 14 (15-19 cm from top; 707.49 mbsf): Mottled background, *Chondrites*, *Planolites*, Clast 15 (27-29 cm from top; 707.61 mbsf): *Planolites*, Clast 16 (52-54 cm from top; 707.86 mbsf): *Planolites*.

364_77_A_081_R_002: Clast 17 (26-34 cm from top; 708.77 mbsf): Mottled background, *Chondrites*, *Planolites*, Clast 18 (37-40 cm from top; 708.86 mbsf): Mottled background, *Planolites*, Clast 19 (52-54 cm from top; 709.01 mbsf): ?*Asterosoma*.

364_77_A_081_R_003: Clast 20 (20-22 cm from top; 710.19 mbsf): ?*Planolites*.

364_77_A_082_R_001: Clast 21 (37-50 cm from top; 710.76 mbsf): Mottled background, *Chondrites*, *Planolites*.

364_77_A_082_R_002: Clast 22 (0-20 cm from top; 710.89 mbsf): Mottled background, *Chondrites*, *Planolites*.

**Supplemental References**

69. Marín, L.E. & Sharpton, V.L. Recent drilling and core recovery within the Chicxulub impact structure, northern Yucatán, Mexico. *EOS trans.* **Suppl**., 408–409 (1994).

70. Marin, L.E., Flores, L.E. & Urrutia-Fucugauchi, J. Perforaciones recientes en el crater de Chicxulub, Yucatán, Mexico. III Reunión Com. Geofís., Inst. Panamericano Geogr. Hist., México, junio 26–30 (1995).

71. Urrutia-Fucugauchi, J., Marin, L. & Trejo, A. UNAM scientific drilling program of Chicxulub impact structure – Evidence for a 300 kilometer crater diameter. *Geophys. Res. Lett.,* **23**, 1565–1568 (1996).

72. Urrutia-Fucugauchi, J., Marin, L. & Trejo, A. Initial results of the UNAM scientific drilling program on the Chicxulub impact structure: rock magnetic properties of UNAM-7 Tekax borehole. *Geofis. Int.* **35**, 125–133 (1996).

73. Rebolledo-Vieyra, M., Urrutia-Fucugauchi, J., Marin, L.E., Trejo-Garcia, A., Sharpton, V.L. & Soler-Arechalde, A.M. UNAM Scientific Shallow-Drilling Program of the Chicxulub Impact Crater. *Int. Geol. Rev.* **42**, 928–940 (2000).

74. Schönian, F.T, Salge, T., Kenkmann, D., Stöffler, A., Soler-Arechalde, A.M. & Urrutia Fucugauchi, L. Chicxulub ejecta blanket: The suevites of the UNAM 5 and UNAM 7 drill cores, Proc. Lunar Planet. Sci. Conf. 37th, Abstract 2229 (2006).

75. Urrutia-Fucugauchi, J., Chávez-Aguirre, J.M., Pérez-Cruz, L. & de la Rosa J.L. Impact ejecta and carbonate sequence in the eastern sector of Chicxulub crater. *Comptes Rend. Geosci.,* **341**, 801–810 (2008).

76. Dressler, B.O., Sharpton, V.L., Morgan, J., Buffler, R., Moran, D., Smit, J., Stöffler, D. & Urrutia-Fucugauchi, J. Investigating a 65-Ma-old smoking gun: Deep drilling of the Chicxulub impact structure. *Eos Transac.* **84**, 125–130 (2003).

77. Dressler, B.O., Sharpton, V.L. & Marin, L.E. Chicxulub Yax-1 impact breccias: Whence they come? (abstract #1259). 34th Lunar and Planetary Science Conference. CD-ROM (2003).

78. Urrutia-Fucugauchi, J., Morgan, J., Stöffler, D. & Claeys, P. The Chicxulub Scienctific Drilling Project (CSDP). *Meteorit. Planet. Sci.* **39**, 787–790 (2004).

79. Whalen, M.T., Gulick, S.P.S., Pearson, Z.F., Norris, R.D., Perez Cruz, L. & Urrutia-Fucugauchi, J. Annealing the Chicxulub impact: Paleogene Yucatàn carbonate slope development in the Chicxulub impact basin, Mexico. In: Verwer, K., Playton, T.E., and Harris, P.M. (Eds.), Deposits, Architecture, and Controls of Carbonate Margin, Slope and Basinal Settings. Special Publication - SEPM (Society for Sedimentary Geology), 105, 282–304 (2013).

80. Kring, D.A., Hôrz, F., Zurcher, L. & Urrutia-Fucugauchi, J. Impact lithologies and their emplacement in the Chicxulub impact crater: Initial results from the Chicxulub Scientific Drilling Project, Yaxcopoil-1, Mexico. *Meteorit. Planet. Sci.* **39**, 879–897 (2004).

81. Velasco-Villareal, M., Urrutia-Fucugauchi, J., Rebolledo-Vieyra, M. & Perez-Cruz, L. Paleomagnetism of impact breccias from the Chicxulub crater – Implications for ejecta emplacement and hydrothermal processes. *Phys. Earth Planet. Inter.,* **186**, 154–171 (2011).

82. Tuchscherer, M.G., Reimold, W.U., Koeberl, C. & Gibson, R.L. Major and trace element characteristics of impactites from the Yaxcopoil-1 borehole, Chicxulub structure, Mexico. *Meteorit. Planet. Sci.* **39**, 955–978 (2004).

83. Smit, J., Claeys, P. & Lowery, C. Isolated deep-water Maastrichtian planktonic foraminifers of the resurge breccia and settling layer of IODP-ICDP Exp364 Hole M0077A in the Chicxulub crater. *Geol. Soc. Amer.* *Abstracts with Programs* **49**,
doi: 10.1130/abs/2017AM-302277 (2017)

84. Dorador, J. & Rodríguez-Tovar, F.J. A novel application of digital image treatment by quantitative pixel analysis to trace fossil research in marine cores. *Palaios* **29**, 533–538 (2014).

85. Dorador, J., Rodríguez-Tovar, F.J. & IODP Expedition 339 Scientists. Quantitative estimation of bioturbation based on digital image analysis. *Mar. Geol.* **349**, 55–60 (2014).

86. Rodríguez-Tovar, F.J. & Dorador, J. Ichnological analysis of Pleistocene sediments from the IODP Site U1385 “Shackleton Site” on the Iberian margin: approaching paleoenvironmental conditions. *Palaeogeogr. Palaeoclimatol. Palaeoecol.* **409**, 24–32 (2014).

87. Dorador, J. & Rodríguez-Tovar, F.J. Application of digital image treatment to the characterization and differentiation of deep-sea ichnofacies. *Spanish J. Palaeontol.* **30**, 265–274 (2015).

88. Dorador, J. & Rodríguez-Tovar, F.J. Stratigraphic variation in ichnofabrics at the “Shackleton Site” (IODP Site U1385) on the Iberian Margin: paleoenvironmental implications. *Mar. Geol.* **377**, 118–126 (2016).

89. Rodríguez-Tovar, F.J. & Dorador, J. Ichnofabric characterization in cores: a method of digital image treatment. *Ann. Soc. Geol. Pol.* **85**, 465–471 (2015).

90. Dorador, J. & Rodríguez-Tovar, F.J. High-resolution image treatment in ichnological core analysis: Initial steps, advances and prospects. *Earth Sci. Rev*. **177**, 226–237 (2018).

91. Pemberton, S.G., MacEachern, J.A., Gingras, M.K. & Bann, K.L. *Atlas of Trace Fossils: The recognition of common trace fossils in outcrop and cores* (Elsevier, 2009).

92. Reineck, H.E. Sedimentgefüge im Bereich der südliche Nordsee. *Abh. Senckenberg. Naturf. Ges.* **505**, 1–138 (1963).

93. Taylor, A.M. & Goldring, R. Description and analysis of bioturbation and ichnofabric. J*. Geol. Soc., London* **150**, 141–148 (1993).

94. Dorador, J., Rodríguez-Tovar, F.J., Mena, A. & Francés, G. Lateral variability of ichnological content in muddy contourites: Weak bottom currents affecting organism’ behaviour. *Sci. Rep*., <https://doi.org/10.1038/s41598-019-54246-3> (2019).

95. King Jr., D.T. & Petruny, L.W., 2020. Chicxulub target stratigraphy and ejecta: insights from northern Belize. *GeoGulf Trans*.**70**, 143–151 (2020).

**Supplementary Tables & Figures**

**Table S1.** Overview of the ichnology, petrography, micropaleontology, and biostratigraphy of the investigated M0077A suevite carbonate clasts. Overview of the ichnology, petrography, micropaleontology, and biostratigraphy of the investigated M0077A suevite carbonate clasts. Biosedimentary structures: *As* = ?*Asterosoma*, *Ch* = *Chondrites*, *Pl* = *Planolites*, *Te* = *Teichichnus*; Bioerosion structures: *Ga* = *Gastrochaenolites*; Biodeformational structures: Mb = Mottled background; pellets = pe.

**Figure S1.** Ichnological features of trace fossil assemblages from Upper Cretaceous clasts, the initial recovery just after the K-Pg, and the early Paleocene sediments. Note variations in Bioturbation Index for trace fossils (BI), ichnodiversity, size of burrows (in mm), and relative abundance. ?*As* = ?*Asterosoma*, Bds = Biodeformational structures, *Ch* = *Chondrites*, *Ga* = *Gastrochaenolites*, *Pa* = *Palaeophycus*, *l* = *Planolites*, *Te* = *Teichichnus*.
