# Supplementary figures and images for: Life before impact in the Chicxulub area: unique marine ichnological signatures preserved in crater suevite
Source: Sci Rep. 2022 Jul 5;12:11376. doi: 10.1038/s41598-022-15566-z (PMC9256630; doi:10.1038/s41598-022-15566-z)

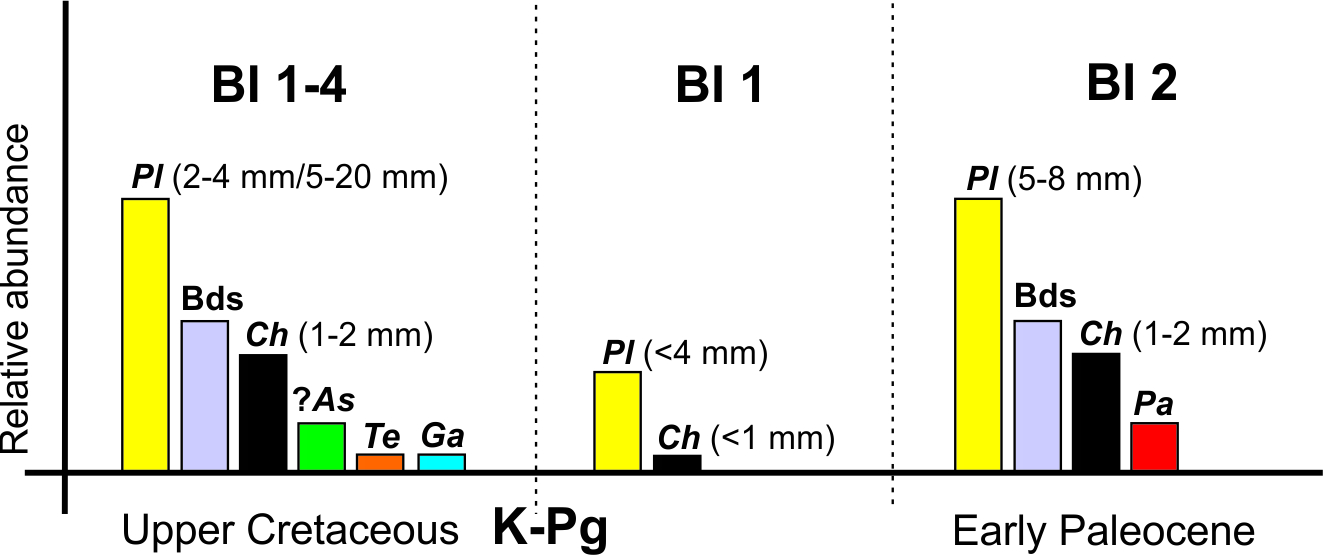

Supplement: Supplementary file 2 — Supplementary Information 2. [file 41598_2022_15566_MOESM2_ESM.jpg]
