# Supplementary material for: Life before impact in the Chicxulub area: unique marine ichnological signatures preserved in crater suevite
Source: Sci Rep. 2022 Jul 5;12:11376. doi: 10.1038/s41598-022-15566-z (PMC9256630; doi:10.1038/s41598-022-15566-z)
Supplement: Supplementary file 3 — Supplementary Information 3. [file 41598_2022_15566_MOESM3_ESM.docx]

**Table S1 Overview of the ichnology, petrography, micropaleontology, and biostratigraphy of the investigated M0077A suevite carbonate clasts. Biosedimentary structures: *As* = ?*Asterosoma*, *Ch* = *Chondrites*, *Pl* = *Planolites*, *Te* = *Teichichnus*; Bioerosion structures: *Ga* = *Gastrochaenolites*; Biodeformational structures: Mb = Mottled background; pellets = pe.**

| **Clast**  **label #** | **Depth (related**  **to core section)** | **Depth**  **(top at mbsf)** | **Ichnotaxa** | **Petrography, micropaleontology, and biostratigraphy** |
| --- | --- | --- | --- | --- |
| 1 | 61_2_49_51 | 679.58 | *Pl*, Mb |  |
| 2 | 61_2_95_96 | 680.04 | *Pl*, *Ch* |  |
| 3 | 61_3_26_28 | 680.76 | *Pl* | Wackestone with indeterminant fossils, one agglutinated benthic foraminifer and *Rugoglobigerina* sp. (Coniacian-Maastrichtian). |
| 4 | 63_3_7_10 | 686.06 | *Pl*, Mb |  |
| 5 | 65_1_79_83 | 688.70 | *Pl*, *Ch*,  ?*As*, Mb | Wackestone rich in planktic foraminifera, these include: *Planoheterohelix* sp. (Cenomanian-Maastrichtian) and keeled foraminifera, most probably *Globotruncana* sp. (Campanian-Maastrichtian). |
| 6 | 73_2_77_80 | 700.69 | *Pl*, *Ch*, Mb |  |
| 7 | 76_1_4_6 | 702.43 | *Te*, *Pl* |  |
| 8 | 76_1_84_87 | 703.23 | *Pl* |  |
| 9 | 76_1_87_90 | 703.26 | pe | Large clast of shallow water carbonate debris rich in larger benthic foraminifera. |
| 10 | 76_1_97_100 | 703.36 | *Pl*, ?*As* |  |
| 11 | 77_1_7_9 | 703.61 | *Ga* | Large dark grey pelagic wackestone. Planktic foraminifera are: *Globotruncanita stuarti* (Campanian-Maastrichtian), *Globigerinelloides* sp. (Valanginian-Maastrichtian), and *Globotruncana* sp. (Coniacian-Maastrichtian). |
| 12 | 78_1_50_52 | 704.24 | *Pl* |  |
| 13 | 78_1_60_63 | 704.34 | *Pl*, *Ch*, Mb |  |
| 14 | 81_1_15_19 | 707.49 | *Pl*, *Ch*, Mb |  |
| 15 | 81_1_27_29 | 707.61 | *Pl* |  |
| 16 | 81_1_52_54 | 707.86 | *Pl* |  |
| 17 | 81_2_26_34 | 708.77 | *Pl*, *Ch*, Mb | Large clast of pelagic packstone with calcispheres, filaments, small benthic foraminifera, *Muricohedbergella* sp. (Albian-Maastrichtian), biserial planktics. Probably late Cenomanian in age. |
| 18 | 81_2_37_40 | 708.86 | *Pl*, Mb |  |
| 19 | 81_2_52_54 | 709.01 | ?*As* | Recrystallized carbonate clast, no recognizable microfossils present. |
| 20 | 81_3_20_22 | 710.19 | ?*Pl* |  |
| 21 | 82_1_37_50 | 710.76 | *Pl*, *Ch*, Mb | Pelagic packstone with calcispheres, poorly preserved biserial planktics (most probably *Planoheterohelix* sp. (Cenomanian-Maastrichtian)) and other indeterminant planktics. |
| 22 | 82_2_0_20 | 710.89 | *Pl*, *Ch*, Mb | Idem as #21 |
